# Silencing of histone deacetylase 3 suppresses the development of esophageal squamous cell carcinoma through regulation of miR-494-mediated TGIF1

**DOI:** 10.1186/s12935-022-02581-3

**Published:** 2022-05-16

**Authors:** Yang Yang, Yuan Zhang, Zongxiang Lin, Kai Wu, Zhanfeng He, Dengyan Zhu, Jia Zhao, Chunyang Zhang, Yuxia Fan

**Affiliations:** 1 Department of Thoracic Surgery, The First Affiliated Hospital of Zhengzhou University, Zhengzhou, 450015 People’s Republic of China; 2 Department of Thyroid Surgery, The First Affiliated Hospital of Zhengzhou University, No. 1, Eastern Jianshe Road, Zhengzhou, 450015 Henan People’s Republic of China

**Keywords:** Histone deacetylase 3, Esophageal squamous cell carcinoma, Transforming growth factor beta-inducing factor 1, microRNA-494, Transforming growth factor-β signaling pathway

## Abstract

**Background:**

Deacetylation of histones by histone deacetylase 3 (HDAC3) acts importantly in modulating apoptosis, DNA damage and cellular progression. Herein, we aimed to unravel the functional role of HDAC3 in a lethal disease, esophageal squamous cell carcinoma (ESCC).

**Methods:**

The expression of HDAC3 in clinically collected ESCC tissues was determined by RT-qPCR and immunohistochemistry. As revealed from bioinformatics analysis, the putative relations between HDAC3 and microRNA-494 (miR-494) and between miR-494 and transforming growth factor beta (TGFβ)-inducing factor 1 (TGIF1) were further verified by chromatin immunoprecipitation and dual-luciferase reporter gene assay. Functional roles of shRNA-mediated depletion of HDAC3, miR-494 mimic and overexpressed TGIF1 were explored by gain- and loss-of-function assays with regard to ESCC cell biological behaviors. A nude mouse model of ESCC was developed for in vivo validation.

**Results:**

HDAC3 was highly expressed in ESCC tissues, suggestive of poor prognosis while TGIF1 was upregulated and miR-494 was downregulated. Mechanistic investigation revealed that HDAC3 inhibited miR-494 expression and TGIF1 was a direct target of miR-494. Furthermore, silencing HDAC3 or overexpressing miR-494 was demonstrated to suppress aggressive phenotypes of ESCC cells both in vitro through the activated TGFβ signaling pathway and in vivo, while TGIF1 overexpression induced opposite results.

**Conclusion:**

Collectively, our findings provided demonstration regarding the oncogenic property of HDAC3 in ESCC via the miR-494/TGIF1/TGFβ axis.

**Supplementary Information:**

The online version contains supplementary material available at 10.1186/s12935-022-02581-3.

## Background

According to GLOBOCAN 2020, esophageal cancer (EC) was reported to be accountable for 604,100 (3.1%) new cases and 544,076 (5.5%) new deaths in 185 countries with about 70% of cases occur in males [1]. Esophageal squamous cell carcinoma (ESCC), the most common histological subtype of EC, is commonly seen in high-incidence areas such as in Central and Southeast Asia [2]. The major risk factors for ESCC are smoking and alcohol intake which exert synergistic effects on carcinogenesis [3]. The clinical management of ESCC is still challenging, and there is currently a lack of approved targeted therapy drugs [4].

Histone deacetylases (HDACs) are capable of inducing the catalyzation of the removal of acetyl groups from the acetyl-lysine residues in histone and non-histone proteins [5]. HDACs are of crucial functionality in various cancers due to their modulation in the function of proteins engaged in cancer initiation and progression [6]. Selective inhibitors for HDACs have been pinpointed as epigenetic drug targets for the treatment of numerous malignancies via diverse molecular mechanisms [7–9]. As a member of the HDACs family, histone deacetylase 3 (HDAC3) has been often activated in several cancers and therefore, its selective inhibitors constitute a preventive strategy against these cancers [10]. HDAC3 has been documented to be highly expressed in ESCC cells, playing a promoting role in tumor progression [11]. As previously demonstrated, HDAC3 was able to limit miR-17-92 expression during lung sacculation [12], highly suggestive of the potential of HDAC3 in modulating microRNAs (miRNAs). miRNAs have been found to exert a tumor suppressive or oncogenic role in EC by mediating their downstream specific target genes [13, 14]. More importantly, overexpression of miR-494 has been elucidated to exert inhibitory effects on malignant features of ESCC cells [15].

Moreover, TGIF1 has been reported to be involved in the onset of ESCC [16]. Notably, TGIF1 has been found to interact with Smad2-Smad4 complex to further block the activation of the TGFβ signaling pathway, which share close correlation with poor prognosis of EC patients and epithelial-mesenchymal transition in ESCC [17–19]. TGFβ signaling pathway is often deregulated in several cancers and possess tumor-suppressor functions in normal cells and early-stage cancer cells [20].

Summarizing the aforementioned available reports, we proposed a hypothesis that aberrantly expressed HDAC3 in EC may participate in the progression and development of EC through regulation of the miR-484/TGIF1/TGFβ signaling. Herein, this study was conducted with purpose of exploring the effect of HDAC3 on EC cellular capacities of proliferation, invasion, migration, and apoptosis by orchestrating miR-484, TGIF1, and the TGFβ signaling pathway.

## Methods

### Ethical statement

This study was approved by the ethics committee of the First Affiliated Hospital of Zhengzhou University with the written informed consent filled by the participating patients. The animal experiment was implemented by referring to the guidelines for the care and use of laboratory animals issued by the National Institutes of Health.

### Bioinformatics analysis

Microarray dataset GSE16456 containing ESCC-related miRNAs was retrieved from the Gene Expression Omnibus (GEO) database, including 6 normal samples and 6 ESCC samples. Differential analysis was then conducted utilizing “limma” package of R language with |log FoldChange|> 1 and *p* value < 0.05 as the threshold. The downstream regulatory miRNAs of HDAC3 were predicted with the RNAInter database. Furthermore, downregulated genes from the GSE16456 dataset and candidate miRNAs were intersected using jvenn. StarBase, mirDIP and miRanda databases were searched for prediction of the downstream target genes of the miRNAs, followed by intersection using jvenn. The expression pattern of genes in ESCC and normal samples available from TCGA was retrieved from the UALCAN database. Kyoto encyclopedia of genes and genomes (KEGG) database was used for analysis of the gene-related signaling pathways.

### Study subjects

ESCC tissues and corresponding adjacent tissues were harvested from 79 patients with ESCC (43 males, 36 females, aged 45–72 years with a mean age of 58.11 ± 8.64) who underwent thoracic surgery at the First Affiliated Hospital of Zhengzhou University from January 2017 to January 2018. Patients were included if (1) their ESCC tissues were resected from surgery at the First Affiliated Hospital of Zhengzhou University; (2) they received no radiotherapy, chemotherapy or immunotherapy before surgery; and (3) their baseline characteristics were complete. Patients were excluded if (1) ESCC was not confirmed by pathobiology; or (2) they had undergone radiotherapy or chemotherapy before surgery. Follow-up started after surgery and lasted for 36 months. The correlation between HDAC3 expression and OS of patients was analyzed with the help of the Kaplan–Meier method.

### Immunohistochemistry

Paraffin-embedded tissue sections were dewaxed, dehydrated and placed in 3% methanol-H_2_O_2_ for 20 min, followed by antigen retrieval. Then, sections were blocked with normal goat serum (C-0005, Shanghai Haoranbio Co., Ltd., Shanghai, China) at ambient temperature for 20 min and reacted with primary antibodies (all from Abcam Inc., Cambridge, MA): rabbit-anti HDAC3 (ab7030, 1: 500,), mouse-anti SMAD2 (ab119907, 1: 200), rabbit-anti SMAD4 (ab40759, 1: 500), rabbit-anti TGF-βRII (ab186838, 1: 200) and rabbit-anti Ki67 (ab15580, 1: 200) at 4 ºC overnight as well as with secondary antibody (Abcam), namely goat anti-rabbit immunoglobulin G (IgG) (ab6721, 1: 2000) or goat anti-mouse IgG (ab150113, 1: 2000) at 37 ºC for 20 min. Subsequently, horseradish peroxidase (HRP)-conjugated streptavidin/peroxidase working solution (0343-10000U, Imunbio Co., Ltd., Beijing, China) was introduced for 20-min of incubation with sections at 37 ºC. The results were visualized using diaminobezidin (ST033, Whiga Co., Ltd., Guangzhou, Guangdong, China). The sections were then counterstained with hematoxylin (PT001, Bogoo Biotech Co., Ltd., Shanghai, China) for 1 min and immersed in 1% ammonia to return blue in color. The mounted sections were microscopically observed with 5 high-power fields selected on a random basis (100 cells each field). The percentage of positive cells < 10% was considered negative and > 50% was considered strongly positive, while 10% ≤ the percentage of positive cells < 50% was considered positive [21].

### Reverse transcription quantitative polymerase chain reaction (RT-qPCR)

Total RNA was extracted utilizing TRIzol reagents (15596026, Invitrogen, Carlsbad, CA, USA), and 1 µg of which was used for RT. For miRNA detection, total RNA was reverse-transcribed into complementary DNA (cDNA) employing miRNA First Strand cDNA Synthesis (Tailing Reaction) kit (B532451-0020, Sangon Biotechnology Co. Ltd., Shanghai, China). For mRNA, RT was started with reference to the manual of cDNA RT kit (K1622, Reanta Co., Ltd., Beijing, China). RT-qPCR was operated on the synthesized cDNA using Fast SYBR Green PCR kit (Applied Biosystems, Foster City, CA, USA) in ABI 7500 RT-qPCR system (Applied Biosystems). Glyceraldehyde-3-phosphate dehydrogenase (GAPDH) or U6 functioned as normalizer and the relative expression of genes was analyzed by the 2^−ΔΔCt^ method. Primer sequences are displayed in Additional file 5: Table S1.

### Western blot analysis

Total protein was extracted followed by concentration measurement utilizing a bicinchoninic acid kit. Protein (50 μg) was separated by sodium dodecyl sulfate–polyacrylamide gel electrophoresis and transferred onto a polyvinylidene fluoride membrane which was then blocked with 5% skimmed milk powder at ambient temperature for 1 h. Next, the membrane was probed with diluted primary rabbit antibodies to Smad2 (ab33875, 1: 1000, Abcam), Smad4 (ab40759, 1: 5000, Abcam), HDAC3 (ab7030, 1: 500, Abcam), TGIF1 (ab52955, 1: 5000, Abcam), cleaved caspase-3 (ab32042, 1: 1000, Abcam), total caspase-3 (ab32150, 1: 1000, Abcam), MMP-2 (ab97779, 1: 2000, Abcam), MMP-9 (ab38898, 1: 1000, Abcam), TGF-βRII (sc-17791, 1: 1000, Santa Cruz Biotechnology, Santa Cruz, CA) and GAPDH (ab9485, 1: 2500, Abcam) overnight at 4ºC. The next day, the membrane was re-probed with HRP-labeled secondary antibody of goat anti-rabbit antibody to IgG H&L (ab97051, 1: 2000, Abcam) for 1 h. The results were visualized utilizing enhanced chemiluminescence reagents (BB-3501, Ameshame, Little Chalfont, UK). Images were acquired through Bio-Rad image analysis system (Bio-Rad Laboratories, Hercules, CA), followed by analysis using Quantity One v4.6.2 software. The relative protein level was described as the ratio of gray value of protein to be tested to that of GAPDH [22, 23].

### Cell treatment

A normal human esophageal epithelial cell (HEEC) line (Tongpai Biotechnology, Shanghai, China) and 4 ESCC cell lines [EC9706 (Fenghui Biotechnology, Changsha, China), Eca109 (Fenghui Biotechnology), TE-1 (Procell, Wuhan, China) and KYSE-150 (Procell)] were cultured in RPMI-1640 medium appended to 10% fetal bovine serum (FBS) and penicillin–streptomycin solution (1: 1) (final concentration of 100 U/mL) at 37ºC with 5% CO_2_. Cells were detached with 0.25% trypsin, passaged (1: 3) and seeded into a 6-well plate (3 × 10^5^ cells/well). Upon achieving 70–80% confluence, cells were collected and cell with the relatively high HDAC3 expression was selected for further experiments.

Logarithmically growing cells with the relatively high HDAC3 expression was passaged, seeded into a 6-well plate (1 × 10^5^ cells/well), and transfected with reference to the manuals of Lipofectamine® 2000 reagent (11668019, Invitrogen) utilizing shRNA against HDAC3 (sh-HDAC3), miR-494 mimic, miR-494 inhibitor, overexpression (oe)-TGIF1, and their corresponding NCs as well as treated with SRI-011381 (an activator of the TGFβ signaling pathway), and dimethyl sulfoxide (DMSO) (negative control [NC] for SRI-011381).

The sequences were provided by Shanghai Sangon. The miRNA mimic transfection concentration was 50 nM, the miRNA inhibitor transfection concentration was 100 nM, and the plasmid transfection amount was 1 μg. For SRI-011381 (HY-100347A, MCE, Sovizzo Vicenza, Italia) treatment, cells after transfection were further treated with 10 μM SRI-011381 for 12 h [24].

### 5-ethynyl-2'-deoxyuridine (EdU) assay

EdU detection kit (Guangzhou RiboBio Co., Ltd., Guangzhou, China) was adopted for testing cell proliferation [25]. Three visual fields were selected on a random basis under a fluorescence microscope (FM-600, Shanghai Pudan Optical Instrument Co., Ltd., Shanghai, China) to count the number of positive cells in each field.

### Flow cytometry

After transfection for 48 h, cells were detached with 0.25% ethylenediaminetetraacetic acid-free trypsin (YB15050057, Yubo Biological Technology Co., Ltd., Shanghai, China) and centrifuged with the supernatant removed. Annexin-V-fluorescein isothiocyanate (FITC)/propidium iodide (PI) dye liquor was formulated using Annexin-V-FITC, PI and 4-(2-hydroxyerhyl)piperazine-1-erhanesulfonic acid (HEPES) at a ratio of 1: 2: 50 with reference to the manual of Annexin-V-FITC cell detection kits (K201-100, Biovision, Milpitas, CA). Each 100 µL dye liquor was adopted to re-suspend 1 × 10^6^ cells and incubated at ambient temperature for 15 min, followed by addition of 1 mL HEPES buffer solution (PB180325, Procell). Finally, cell apoptosis was assessed by detecting FITC and PI fluorescence utilizing a flow cytometer (FACS Calibur, BD Biosciences, Franklin Lakes, NJ, USA) [26, 27].

### Transwell assay

The cell migratory and invasive (added with extracellular matrix gel) capabilities were examined using a Transwell assay, as previously described [28]. Five randomly selected fields in each chamber were observed, photographed, and counted under an inverted microscope.

### Chromatin immunoprecipitation (ChIP) assay

Fresh ESCC and adjacent tissues were cut into 1–3 mm^3^ small pieces and transferred to a 50 mL test tube. Next, the tissues were fixed with formaldehyde at ambient temperature for 10 min for generating DNA–protein crosslink. The reaction was terminated by 2.5 M Glycine (final concentration of 0.125 M). The samples were lysed using a homogenizer, sub-packed (1 mL per tube), re-suspended, and fragmented by ultrasonic wave, 10 s each at an interval of 10 s, 15 cycles in total. The supernatant was harvested through centrifugation at 13,000 rpm and 4ºC for 10 min and sub-packed into 3 tubes. The tubes were respectively added with normal mouse antibody to IgG (ab109489, 1: 100, Abcam) as NC and mouse antibody to HDAC3 (ab7030, 1: 100, Abcam) for incubation at 4ºC overnight. The endogenous DNA–protein complex was precipitated by Protein Agarose/Sepharose. The non-specific complex was washed and the DNA–protein complex was incubated at 65ºC overnight for de-crosslinking. DNA fragment was purified and retrieved by phenol/chloroform. The primer was designed by selecting 2000 bp from the transcription start site to the upstream as the promoter sequence. The enrichment of HDAC3 in the miR-494 promoter region was tested by RT-qPCR [29].

### Dual luciferase reporter gene assay

The HEK-293T cell line (American Type culture collection, Manassas, VA) was cultured in DMEM. When achieving 80–90% confluence, cells were detached by 0.25% trypsin, passaged and cultured at 37 ºC with 5% CO_2_. Logarithmically growing cells were used for further experiments. The artificially synthesized TGIF1 3’untranslated region (3’UTR) was introduced into pGL3-control (Promega Corp., Madison, WI) through endonuclease sites XhoI and BamH I. The mutation site of complementary sequence of seed sequence was designed on TGIF1-wild-type (WT). After restriction enzyme digestion, the target fragment was inserted into pGL3-control vector using T4 DNA ligase. The correctly sequenced luciferase reporter plasmids WT and mutant type (MUT) were co-transfected with miR-494 mimic/mimic-NC into HEK-293T cells, respectively, for 48 h. The luciferase activity was tested utilizing Dual-Luciferase Reporter Assay System Kit (Promega) on a TD-20/20 luminometer (E5311, Promega) [30].

### Xenograft tumor in nude mice

In total, 36 thymic male nude mice aged 5 to 6 weeks (Shanghai SLAC Laboratory Animal Co., Ltd., Shanghai, China) were housed at 25–27 ºC under constant humidity (60–65%) under 12-h light/dark cycles (drink and eat freely). The animal experiment started after one week of acclimation. Under 80–90% confluence, EC9706 and Eca109 cells transfected with sh-NC + oe-NC, sh-HDAC3 + oe-NC or sh-HDAC3 + oe-TGIF1 were collected, detached, centrifuged, re-suspended and counted. The cell concentration was adjusted to 1 × 10^7^ cells/mL. Then, 20 µL cell suspension was subcutaneously inoculated into the armpit of nude mice (n = 6). The tumor formation was observed every week. Tumor volume (V) was calculated: V = (a _longest diameter_ × b _shortest diameter_^2^)/2, followed by construction of a tumor growth curve. All nude mice were euthanized utilizing CO_2_ after 5 weeks.

### Hematoxylin and eosin (HE) staining

HE staining kits (C0105, Beyotime, Shanghai, China) were used for the staining. In brief, the subcutaneously transplanted tumor tissues were fixed with 10% neutral buffered formaldehyde (G2161, Solarbio, Beijing, China) at 4 ºC for 24 h, The tissues were then dehydrated, paraffin-embedded and sectioned, conventionally dewaxed with xylene and hydrated with gradient alcohol. Hematoxylin was used to stain the sections for 5–10 min, followed by eosin staining solution for 30 s-2 min. Finally, the sections were dehydrated with gradient alcohol, cleared with xylene, sealed with neutral gum, photographed and observed under an inverted microscope (Olympus, IX73) [31].

### Statistical analysis

Statistical analyses were started with the help of SPSS 21.0 (IBM Corp., Armonk, NY, USA). All experimental data were tested for normal distribution and homogeneity of variance. Measurement data were expressed as mean ± standard deviation. Differences between ESCC and adjacent tissues were compared by paired *t* test while data comparison of unpaired design between other two groups was conducted by independent sample *t* test. Differences among multiple groups were compared by one-way analysis of variance (ANOVA) in combination with Tukey’s post hoc test. Data at various time points were compared by repeated measures ANOVA with post-hoc Bonferroni-corrected comparisons. The survival rate of patients was calculated by the Kaplan–Meier method. A value of *p* < 0.05 was summarized as statistically significant.

## Results

### Elevated HDAC3 in ESCC positively correlated with poor prognosis

**Fig. 1:**
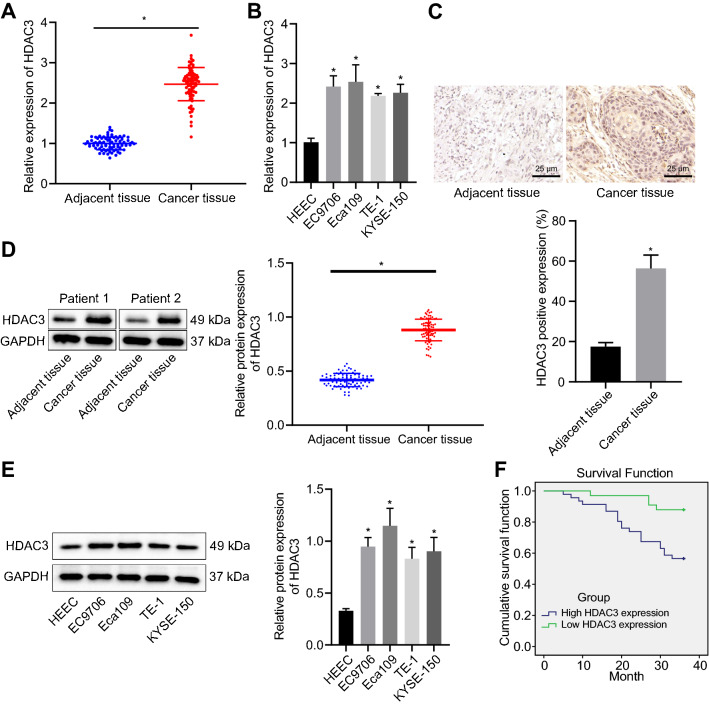
High expression of HDAC3 is indicative of poor prognosis in patients with ESCC. **A** HDAC3 expression in ESCC and adjacent tissues (n = 79). **B** HDAC3 expression in HEECs and ESCC cell lines (EC9706, Eca109, EC1 and KYSE-150). **C** Immunohistochemistry analysis of HDAC3 protein in representative ESCC and adjacent tissues (scale bar: 25 μm). **D** HDAC3 protein expression in ESCC and adjacent tissues (n = 79). **E** HDAC3 protein expression in HEECs and EC cell lines (EC9706, Eca109, EC1 and KYSE-150). **F** Correlation between HDAC3 expression and OS of patients with ESCC (n = 79). * *p* < 0.05 vs*.* adjacent tissues, HEECs or patients with low expression of HDAC3. Measurement data were expressed as mean ± standard deviation derived from at least 3 independent experiments. Data between ESCC and adjacent tissues were compared by paired *t* test. Data comparison among multiple groups was conducted by one-way ANOVA, followed by Tukey’s post hoc test. Survival rate of patients was calculated by Kaplan–Meier method and survival difference was tested by log-rank

Firstly, as described by RT-qPCR, significantly higher expression of HDAC3 was detected in ESCC tissues (Fig. 1A). Higher expression of HDAC3 was also observed in ESCC cell lines, EC9706, Eca109, EC1 and KYSE-150 compared with HEECs, of which EC9706 and Eca109 cells showed more pronounced elevation (Fig. 1B) and therefore were selected for subsequent experiments. Furthermore, immunohistochemistry showed the same expression tendency as RT-qPCR and that HDAC3 was mainly expressed in the nucleus (Fig. 1C). In addition, protein expression of HDAC3 showed same changing trend as mRNA expression in ESCC tissues and ESCC cell lines, as confirmed by Western blot assay (Fig. 1D, 1E). Correlation analysis between expression pattern of HDAC3 and clinicopathological data of patients revealed no correlation of HDAC3 expression with age or gender. However, a significant positive correlation was noted between HDAC3 expression and tumor node metastasis staging, lymph node metastasis (LNM) and tumor size (Additional file 5: Table S2). Analysis using the Kaplan–Meier method suggested that patients with upregulated HDAC3 expression depicted significantly shorter overall survival than those with downregulated HDAC3 expression (Fig. 1F), suggesting that highly expressed HDAC3 was related to poor prognosis. Taken together, these results indicated that upregulation of HDAC3 in ESCC was correlated with dismal prognosis of patients with ESCC.

### Silencing of HDAC3 curbed ESCC cell malignant properties

**Fig. 2:**
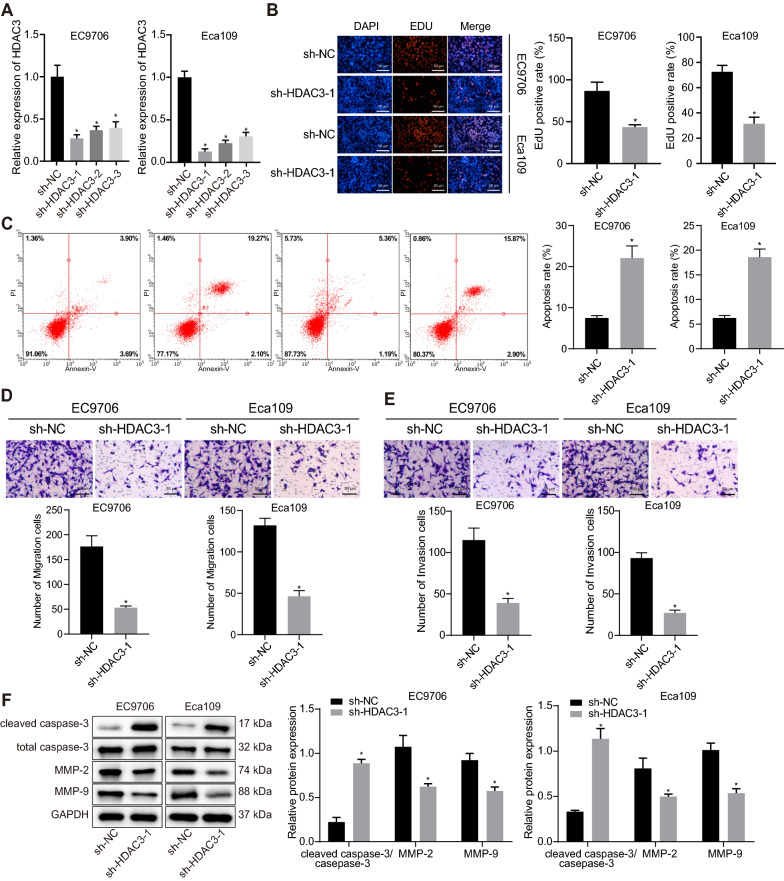
shRNA-mediated silencing of HDAC3 arrests proliferation, migration and invasion of ESCC cells but triggers apoptosis. **A** HDAC3 expression in EC9706 and Eca109 cells transfected with sh-HDAC3. **B** Proliferation of EC9706 and Eca109 cells transfected with sh-HDAC3, scale bar = 50 μm. **C** Apoptosis of EC9706 and Eca109 cells transfected with sh-HDAC3. **D** Migration of EC9706 and Eca109 cells transfected with sh-HDAC3 (scale bar: 50 μm). **E** Invasion of EC9706 and Eca109 cells transfected with sh-HDAC3 (scale bar: 50 μm). **F** Protein levels of cleaved caspase-3, total caspase-3, MMP-2 and MMP-9 normalized to GAPDH in EC9706 and Eca109 cells transfected with sh-HDAC3. * *p* < 0.05 vs. EC9706 and Eca109 cells transfected with sh-NC. Measurement data were expressed as mean ± standard deviation derived from at least 3 independent experiments. Data between two groups were compared by unpaired *t* test. Data comparison among multiple groups was conducted by one-way ANOVA, followed by Tukey’s post hoc test

With results determining the aberrant expression pattern of HDAC3 in ESCC, HDAC3 expression was then silenced in EC9706 and Eca109 cells for exploration on the resultant cellular biological functions. sh-HDAC3-1 with highest silencing efficiency was selected for subsequent experiments as detected by RT-qPCR (Fig. 2A). Additionally, cell proliferative, migratory, and invasive were potentials significantly curbed but apoptosis was promoted in response to sh-HDAC3-1 (Fig. 2B–E). Meanwhile, Western blot analysis depicted that the protein expression of matrix metalloprotein-2 (MMP)-2 and MMP-9 was downregulated while that of cleaved caspase-3/total caspase-3 was upregulated in EC9706 and Eca109 cells in the presence of sh-HDAC3-1 (Fig. 2F). To conclude, these findings offered evidence on the anti-tumor action of HDAC3 silencing by inhibiting malignant cellular properties of ESCC cells.

### HDAC3 silencing repressed ESCC cell malignant features by upregulating miR-494

**Fig. 3:**
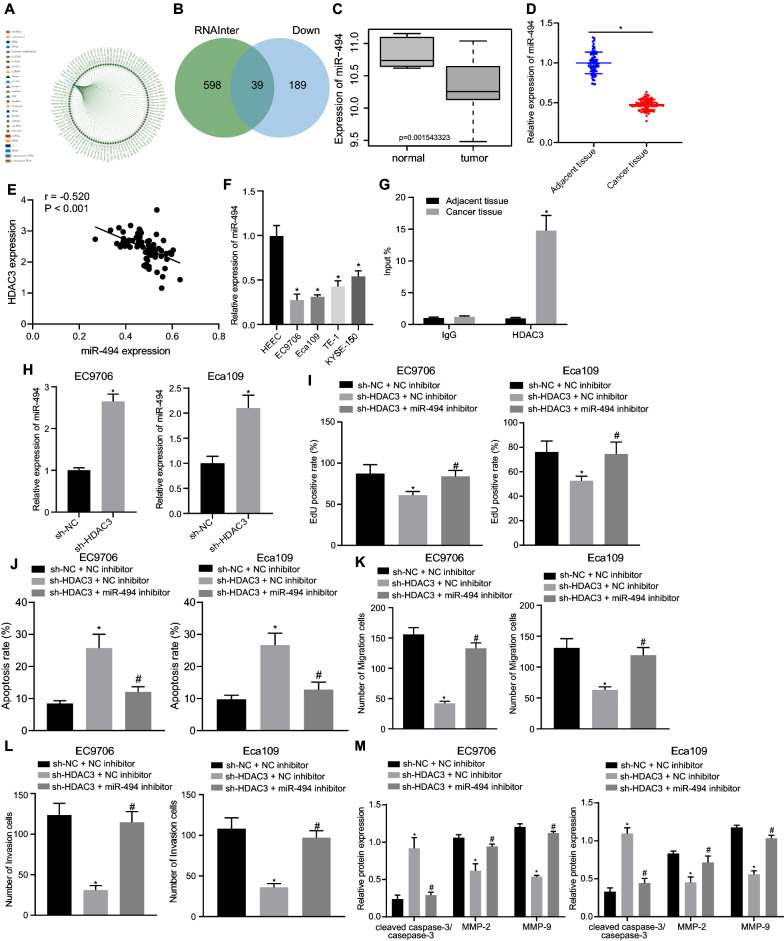
HDAC3 downregulates miR-494 expression by enriching in the miR-494 promoter to promote ESCC cell malignant phenotypes. **A** Venn diagram displaying the intersection of downstream miRNAs for HDAC3 predicted by the RNAInter database. **B** Venn diagram displaying the intersection of candidate miRNAs and 228 downregulated genes in the GSE16456 dataset. **C** A box plot displaying miR-494 expression in the GSE16456 dataset. **D** miR-494 expression in ESCC and adjacent tissues (n = 79). **E** Pearson analysis for the correlation between HDAC3 and miR-494 expression. **F** HDAC3 protein expression in HEECs and EC cell lines (EC9706, Eca109, EC1 and KYSE-150). **G** Enrichment of HDAC3 in the miR-494 promoter region in ESCC and adjacent tissues. **H** miR-494 expression in EC9706 and Eca109 cells transfected with sh-HDAC3. **I** Proliferation of EC9706 and Eca109 cells transfected with sh-HDAC3 or in combination with miR-494 inhibitor. **J** Apoptosis of EC9706 and Eca109 cells transfected with sh-HDAC3 or in combination with miR-494 inhibitor. **K** Migration of EC9706 and Eca109 cells transfected with sh-HDAC3 or in combination with miR-494 inhibitor. **L** Invasion of EC9706 and Eca109 cells transfected with sh-HDAC3 or in combination with miR-494 inhibitor. **M** Protein levels of cleaved caspase-3, total caspase-3, MMP-2 and MMP-9 normalized to GAPDH in EC9706 and Eca109 cells transfected with sh-HDAC3 or in combination with miR-494 inhibitor. *p < 0.05 vs. adjacent tissues, HEECs or EC9706 and Eca109 cells transfected with sh-NC or sh-NC + NC inhibitor. #*p* < 0.05 vs*.* EC9706 and Eca109 cells transfected with sh-HDAC3 + NC inhibitor. Measurement data were expressed as mean ± standard deviation derived from at least 3 independent experiments. Data between ESCC and adjacent tissues were compared by paired *t* test while data comparison between other two groups was analyzed by unpaired *t* test. Data comparison among multiple groups was conducted by one-way ANOVA, followed by Tukey’s post hoc test

RNAInter website was introduced to predict the downstream regulators of HDAC3. As a result, 637 miRNAs that interacted with HDAC3 were identified, and interaction networks of top 100 miRNAs with higher degree of confidence are shown in Fig. 3A. Differential analysis on the microarray dataset GSE16456 revealed 228 downregulated miRNAs, and 39 candidate miRNAs were further found in the intersection (Fig. 3B). In addition, the microarray dataset GSE16456 showed that miR-494 was poorly expressed in ESCC (Fig. 3C). Therefore, it was inferred that miR-494 might participate in the development and progression of ESCC. RT-qPCR results revealed significantly lower expression of miR-494 in ESCC tissues (Fig. 3D). Besides, miR-494 expression showed negative correlation with HDAC3 expression (Fig. 3E). Similarly, compared with HEEC, low expression of miR-494 was also observed in ESCC cell lines, EC9706, Eca109, EC1, and KYSE-150, whilst much more significant differences were detected in EC9706 and Eca109 cells (Fig. 3F). The enrichment of HDAC3 in the miR-494 promoter region was detected by ChIP assay, which revealed that HDAC3 was significantly enriched in the miR-494 promoter region in ESCC tissues than that in adjacent tissues (Fig. 3G). Further RT-qPCR test noted that miR-494 expression was elevated by sh-HDAC3 in EC9706 and Eca109 cells (Fig. 3H). Subsequently, we found that cell proliferative, migratory and invasive capacities were obviously inhibited while apoptosis was enhanced in the presence of sh-HDAC3, action of which was counterweighed by further delivery of miR-494 inhibitor (Fig. 3I–L, Additional file 1: Figure S1A–D). Western blot analysis revealed that sh-HDAC3 caused a significant downregulation of MMP-2 and MMP-9 and upregulation of cleaved caspase-3/total caspase-3 which was further reversed by miR-494 inhibitor in EC9706 and Eca109 cells (Fig. 3M, Additional file 1: Figure S1E). To sum up, the oncogenic role of HDAC3 in ESCC was demonstrated to be dependent on miR-494 inhibition.

### MiR-494 inhibited ESCC cell malignant behaviors by targeting TGIF1

**Fig. 4:**
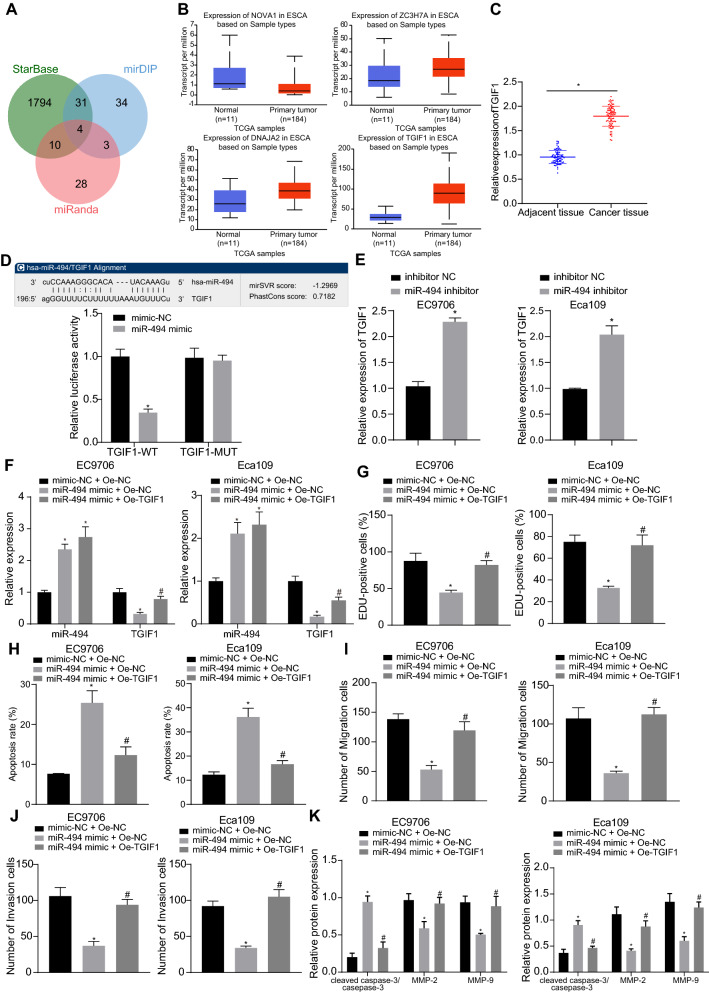
Restored miR-494 downregulates TGIF1 expression to inhibit ESCC cell malignant phenotypes. **A** Venn diagram displaying the intersection of target genes of miR-494 from StarBase, mirDIP and miRanda databases. **B** A box plot displaying expression of 4 candidate genes (NOVA1, ZC3H7A, DNAJA2 and TGIF1) in ESCC and normal samples from the UALCAN database. **C** Expression of TGIF1 in ESCC tissues and adjacent tissues detected by RT-qPCR. n = 79. *p < 0.05 vs. adjacent tissues. **D** Binding of miR-494 to TGIF1 confirmed by dual-luciferase reporter gene assay in HEK-293T cells. *p < 0.05 vs*.* the HEK-293T cells transfected with mimic NC. **E** TGIF1 expression in EC9706 and Eca109 cells transfected with miR-494 inhibitor. *p < 0.05 vs*.* cells transfected with inhibitor NC. **F** Expression of miR-494 and TGIF1 in EC9706 and Eca109 cells transfected with miR-494 mimic or in combination with oe-TGIF1. **G** EC9706 and Eca109 cell proliferation following transfection with miR-494 mimic or in combination with oe-TGIF1. **H** EC9706 and Eca109 cell apoptosis following transfection with miR-494 mimic or in combination with oe-TGIF1. **I** EC9706 and Eca109 cell migration following transfection with miR-494 mimic or in combination with oe-TGIF1. **J** EC9706 and Eca109 cell invasion following transfection with miR-494 mimic or in combination with oe-TGIF1. **K** Protein levels of cleaved caspase-3, total caspase-3, MMP-2 and MMP-9 normalized to GAPDH in EC9706 and Eca109 cells transfected with miR-494 mimic or in combination with oe-TGIF1. In Panel F-K, * *p* < 0.05 vs. cells transfected with mimic-NC + oe-NC. #*p* < 0.05 vs*.* cells transfected with miR-494 mimic + oe-NC. Measurement data were expressed as mean ± standard deviation derived from at least 3 independent experiments. Data between ESCC and adjacent tissues were compared by paired *t* test while data comparison between other two groups was analyzed by unpaired *t* test. Data comparison among multiple groups was conducted by one-way ANOVA, followed by Tukey’s post hoc test

According to further prediction regarding downstream target genes of miR-494, 4 candidate genes, NOVA1, ZC3H7A, DNAJA2 and TGIF1, were obtained at the intersection of results from StarBase, mirDIP and miRanda (Fig. 4A). The expression patterns of the 4 candidate genes in ESCC and normal samples of The Cancer Genome Atlas (TCGA) were then retrieved in UALCAN, and results revealed high expression of TGIF1 with the most significant difference in ESCC samples (Fig. 4B). Similar elevation was validated by RT-qPCR (Fig. 4C). The putative binding sites between miR-494 and TGIF1 were analyzed by the miRanda website. Dual-luciferase reporter gene assay for verification purpose showed that miR-494 mimic significantly weakened the luciferase activity of TGIF1-WT yet no significant difference was witnessed regarding the luciferase activity of TGIF1-MUT (Fig. 4D). Moreover, TGIF1 expression in EC9706 and Eca109 cells was found to be increased by miR-494 inhibitor (Fig. 4E). The above findings verified the targeting relation between miR-494 and TGIF1.

After cell transfection, the efficiency was evaluated by RT-qPCR, results of which showed that miR-494 expression was significantly elevated and TGIF1 expression was reduced by miR-494 mimic, while further transduction of oe-TGIF1 exerted insignificant effect on miR-494 expression but increased TGIF1 expression (Fig. 4F). Furthermore, as revealed from EdU assay (Fig. 4G, Additional file 2: Figure S2A), flow cytometry (Fig. 4H, Additional file 2: Figure S2B) and Transwell assay (Fig. 4I, j, Additional file 2: Figure S2C, D), the suppressive action of miR-494 mimic on EC9706 and Eca109 cell proliferation, migration and invasion and its promoting action on cell apoptosis were abolished by overexpressing TGIF1. Western blot analysis showed that in EC9706 and Eca109 cells, significant downregulation of MMP-2 and MMP-9 and upregulation of cleaved caspase-3/total caspase-3 induced by miR-494 mimic were also restored by overexpressing TGIF1 (Fig. 4K, Additional file 2: Figure S2E). Therefore, a conclusion could be drawn that the anti-tumor action of miR-494 in ESCC relied on TGIF1 downregulation.

### TGIF1 promoted ESCC cell malignant behaviors by inactivating the TGFβ signaling pathway

**Fig. 5:**
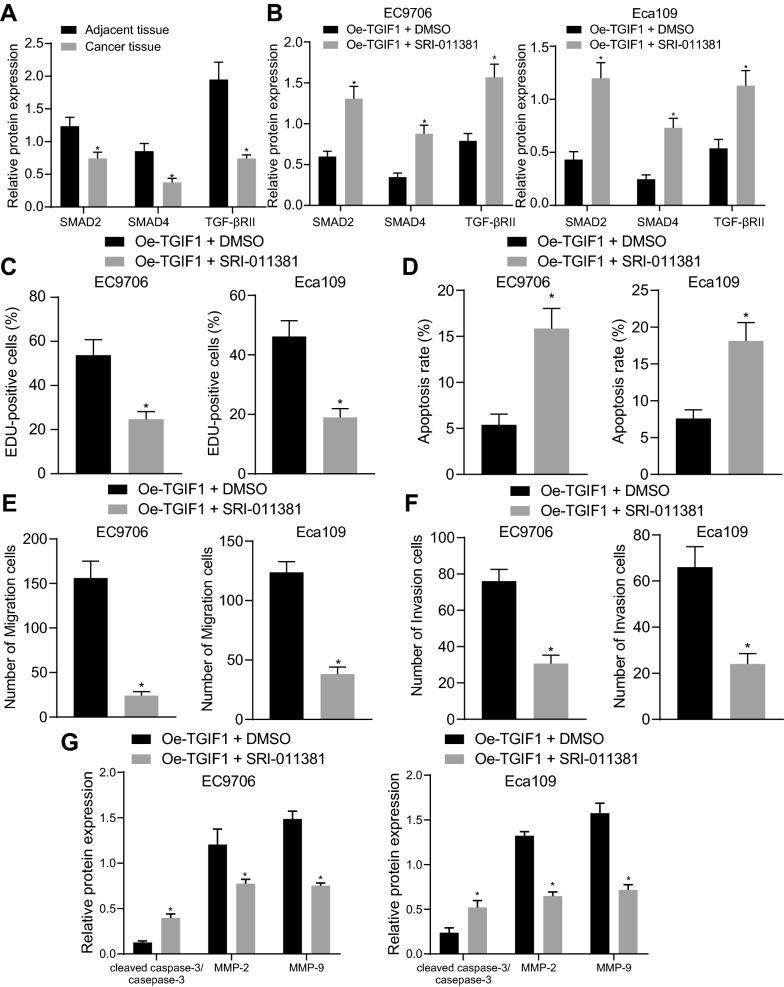
TGIF1 inactivates the TGFβ signaling pathway to enhance ESCC cell malignant phenotypes. **A** Expression of the TGFβ signaling pathway-related proteins (Smad2, Smad4 and TGF-βRII) in ESCC and adjacent tissues normalized to GAPDH. n = 79. * *p* < 0.05 vs*.* adjacent tissues. **B** Expression of the TGFβ signaling pathway-related proteins (Smad2, Smad4 and TGF-βRII) in EC9706 and Eca109 cells treated with oe-TGIF1 or in combination with SRI-011381 normalized to GAPDH. **C** Proliferation of EC9706 and Eca109 cells treated with oe-TGIF1 or in combination with SRI-011381. **D** Apoptosis of EC9706 and Eca109 cells treated with oe-TGIF1 or in combination with SRI-011381. **E** Migration of EC9706 and Eca109 cells treated with oe-TGIF1 or in combination with SRI-011381. **F** Invasion of EC9706 and Eca109 cells treated with oe-TGIF1 or in combination with SRI-011381. **G** Protein levels of cleaved caspase-3, total caspase-3, MMP-2 and MMP-9 normalized to GAPDH in EC9706 and Eca109 cells treated with oe-TGIF1 or in combination with SRI-011381. In Panel B-G, *p < 0.05 vs. EC9706 and Eca109 cells treated with oe-TGIF1 + DMSO. #*p* < 0.05 vs. EC9706 and Eca109 cells treated with oe-TGIF1 + SRI-011381. Measurement data were expressed as mean ± standard deviation derived from at least 3 independent experiments. Data between ESCC and adjacent tissues were compared by paired *t* test while data comparison between other two groups was analyzed by unpaired *t* test

It was found that TGIF1 inhibited activation of the TGFβ signaling pathway after searching KEGG database (map04350). Western bot analysis was operated to measure the expression of TGFβ signaling pathway-related factors (Smad2, Smad4, and TGF-βRII), which were revealed to be diminished in ESCC tissues in comparison to adjacent tissues (Fig. 5A, Additional file 3: Figure S3A). After addition of SRI-011381, an activator of the TGFβ signaling pathway, expression of Smad2, Smad4, and TGF-βRII was elevated in response to treatent of oe-TGIF1 as detected by Western blot analysis (Fig. 5B, Additional file 3: Figure S3B). Additionally, treatment of both oe-TGIF1 and SRI-011381 corresponded to suppressed cell proliferation, migratory, and invasive capacities and promoted apoptosis accompanied by diminished levels of MMP-2 and MMP-9 and elevated levels of cleaved caspase-3/total caspase-3 (Fig. 5C–G, Additional file 3: Figure S3C-G). These results highlighted the involvement of inactivation of the TGFβ signaling pathway in the oncogenic role of TGIF1 in ESCC.

### Silencing of HDAC3 inhibited tumor growth of ESCC in vivo via the MiR-494/TGIF1/TGFβ axis.

**Fig. 6:**
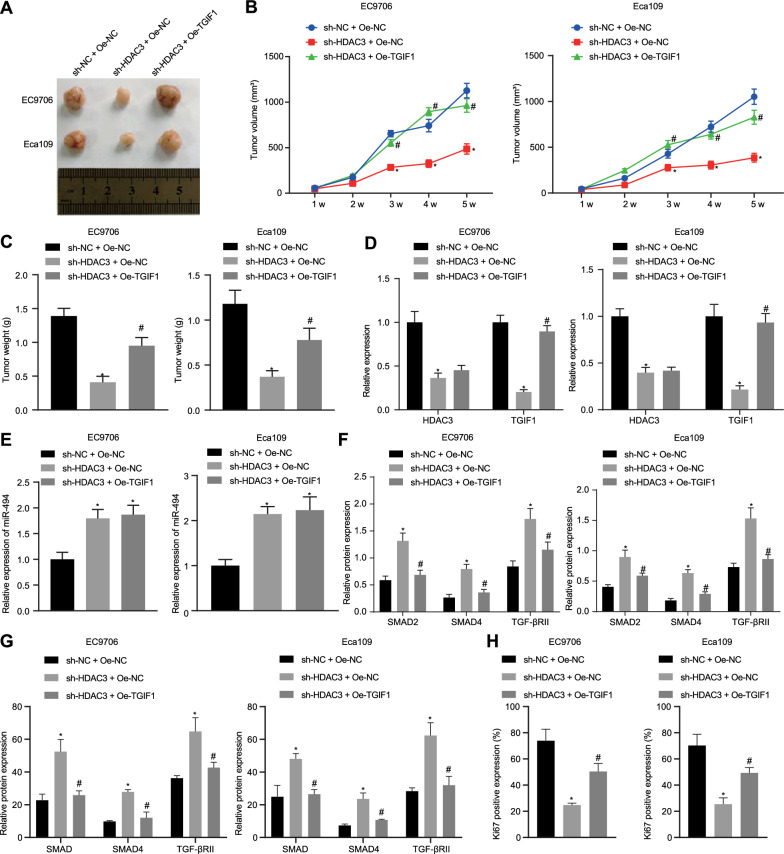
shRNA-mediated silencing of HDAC3 suppresses tumor growth of ESCC in vivo through the miR-494/TGIF1/TGFβ axis. **A** White light images showing xenografts in nude mice. **B** Tumor volume of nude mice. **C** Tumor weight of nude mice. **D** Expression of HDAC3 and TGIF1 in tumor tissues of nude mice. **E** miR-494 expression in tumor tissues. **F** Expression of the TGFβ signaling pathway-related proteins (Smad2, Smad4 and TGF-βRII) in tumor tissues detected by Western blot analysis, normalized to GAPDH. **G** Expression of the TGFβ signaling pathway-related proteins (Smad2, Smad4 and TGF-βRII) in tumor tissues detected by Immunohistochemistry. **H** Expression of Ki67 in tumor tissues of nude mice and the pathological conditions of tumor tissues. n = 6. *p < 0.05 vs. nude mice harboring cells transfected with sh-NC + oe-NC. #*p* < 0.05 vs. nude mice harboring cells transfected with sh-HDAC3 + oe-NC. Measurement data were expressed as mean ± standard deviation derived from at least 3 independent experiments. Data comparison among multiple groups was conducted by one-way ANOVA, followed by Tukey’s post hoc test. Tumor volume was analyzed by Bonferroni-corrected repeated measures ANOVA

Lastly, the aforementioned findings were validated in vivo. It was observed that tumor weight was much lighter and tumor growth speed was slower in the presence of sh-HDAC3, which was negated by oe-TGIF1 treatment (Fig. 6A–C). RT-qPCR and Western blot analysis revealed that the expression of HDAC3 and TGIF1 was downregulated while that of Smad2, Smad4 and TGF-βRII was upregulated in response to sh-HDAC3; these effects could be reversed by oe-TGIF1 treatment (Fig. 6D–F, Additional file 4: Figure S4A). Similar changing tendency was observed after immunohistochemistry as from Western blot results (Fig. 6G, Additional file 4: Figure S4B).

Furthermore, immunohistochemistry and HE staining showed that the expression of Ki67 and tumor cells in nude mice were diminished in the presence of sh-HDAC3, the results of which could be reversed by further oe-TGIF1 treatment (Fig. 6H, Additional file 4: Figure S4C).

Collectively, the tumor suppressive activity of HDAC3 silencing was reproduced in vivo dependent on the miR-494/TGIF1/TGFβ axis.

## Discussion

More patients with EC can only be diagnosed at an advanced stage yet the potency of traditional radiotherapy or chemotherapy is limited; therefore, a better understanding of underlying molecular mechanism may provide future direction of developing novel therapeutic strategies [32]. During the present study, we attempted to unravel the involvement of HDAC3 in the context of ESCC. Collectively, the experimental data in our study offered evidence that shRNA-mediated silencing of HDAC3 harbored the tumor suppressive property to curb proliferative, migratory and invasive capabilities of ESCC cells and to induce apoptotic process through activation of the TGFβ signaling pathway via target-inhibition of TGIF1 by miR-494.

HDAC3 has been highlighted to be a crucial validated target for cancer due to its overexpression in a variety of cancers [10]. Our study underlined the identification of upregulated HDAC3 expression in ESCC tissues and cells. In line with our findings, esophageal tumor tissues showed upregulation of HDAC3 as compared to normal adjacent tissues, and conversely, its inhibition contributes to the arrested malignant properties of EC cells [11]. When HDAC3 was silenced in our study, malignant behaviors of ESCC cells were restrained yet apoptosis was accelerated accompanied by upregulated cleaved caspase-3/total caspase-3 and downregulated MMP-2 and MMP-9. Activation of caspase-3 has been used as a hallmark of induced apoptosis in EC cells [33]. Highly expressed MMP-9 has been found in EC tissues, and is associated with poor prognosis, tumor size and clinical staging [34, 35]. In the context of ESCC, a high positive rate of MMP-2 has been detected in relation to tumor differentiation degree, invasion depth and LNM so that occurrence of distant metastasis is facilitated [36]. Notably, HDAC3 has been shown to augment expression of MMP-2 in U87MG cells [37] but suppress caspase-3 expression in K562 cells [38]. Therefore, strategies aimed at HDAC3 silencing could prove beneficial for the treatment of ESCC.

An inverse correlation has been confirmed between HDAC3 expression and miR-494 expression in the context of acute ischemic stroke [39]. Consistently, the current results revealed that HDAC3 played an inhibitory role in miR-494 expression by enriching in its promoter. Moreover, miR-494, in the present study was found to be underexpressed while TGIF1 was overexpressed in ESCC tissues and cells. In accordance to this finding, downregulation of miR-494 has been identified in EC by multiple functional reports [15, 40, 41]. However, overexpression of miR-494 harbors the potential to curb the proliferative and invasive capabilities of EC cells and to trigger apoptosis by targeting CLPTM1L [15]. Meanwhile, in the current study, the anti-oncogenic property of miR-494 could be abolished by overexpression of its target gene, TGIF1. miRNAs can interact with the 3’UTR of specific target mRNAs and ultimately cause the repression of their expression [42]. In this study, we also confirmed that miR-494 bound to the 3’UTR of TGIF1 mRNA and could negatively regulate its expression. TGIF1 is able to encourage non-small cell lung cancer cells to grow and migrate while TGIF1 knockdown ameliorates tumorigenic characteristics [43]. Additionally, the tumor-promoting action of TGIF1 has been discussed in papillary thyroid cancer where downregulation of TGIF1 limits the progression of malignant cells in vitro and suppresses aggressive phenotypes in vivo [44]. However, the expression and function of TGIF1 in ESCC have been not fully elucidated. Additionally, owing to the scarcity of literature on the relationship between HDAC3 and TGIF1, further investigation is still required so as to validate the established promoting effect of HDAC3 on ESCC cell malignant phenotypes and tumor growth through blockade of miR-494-mediated TGIF1 inhibition.

In the present investigation, we also found that activation of the TGFβ signaling pathway could be inhibited by TGIF1, facilitating the malignant phenotypes of ESCC cells. The prominent function of TGIF1 has been indicated as the suppression of the TGFβ signaling pathway in the progression of pancreatic ductal adenocarcinoma [45]. TGIF1 can bind to DNA through interplay with Smad2 in the TGFβ signaling pathway and subsequently inhibit the activation of downstream genes of the TGFβ signaling pathway [17]. TGFβ signaling pathway has been frequently deregulated in tumors and exerts crucial roles in tumor initiation, development and metastasis [46]. More importantly, blockade of the TGFβ signaling pathway has been elaborated to accelerate the progression of EC [47], supporting the validation of our results. In addition, TGFβ1 has been found to be a major factor inducing the upregulation of miR-494 in myeloid-derived suppressor cells [48]. A previous work also indicated that HDAC3 deficiency contributes to the promotion of TGFβ signaling pathway activation in PM2.5-induced mice with lung injury [49]. Based on the aforementioned information, we are convinced that the use of a TGFβ signaling pathway activator may lead to a new therapeutic strategy in patients with ESCC (Additional file 6).

## Conclusion

**Fig. 7:**
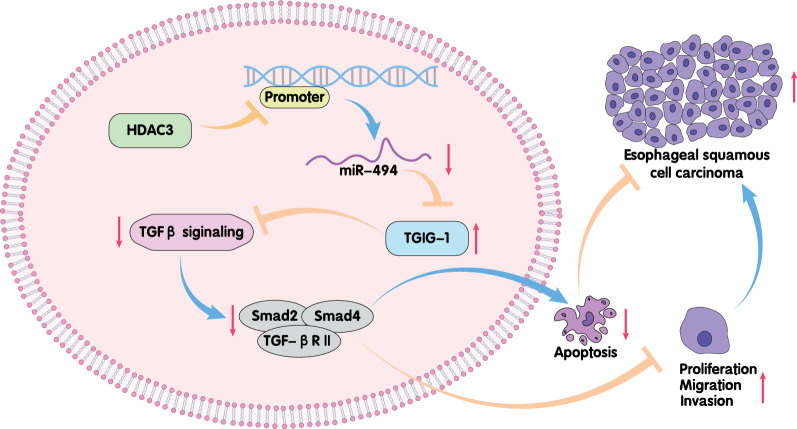
Potential role of HDAC3/miR-494/TGIF1/TGFβ axis in ESCC. shRNA-mediated silencing of HDAC3 harbored the tumor suppressive property to curb proliferative, migratory and invasive capabilities of ESCC cells and to induce apoptotic process through activation of the TGFβ signaling pathway via target-inhibition of TGIF1 by miR-494

To conclude, our findings demonstrate that HDAC3 silencing can alleviate ESCC cells malignant properties by upregulating miR-494 to limit TGIF1 expression, the mechanism of which was dependent on the activated TGFβ signaling pathway (Fig. 7). However, due to the currently limited experimental conditions, only the interaction between HDAC3 and TGIF1 was investigated in the nude mouse model of ESCC, which requires further research for validation of the reported signal axis in vivo. Moreover, since therapeutic approaches referring miRNAs remain in their infancy, our results are quite encouraging and underline that miR-494 can be targeted to develop a novel treatment modality in patients with ESCC, providing a prospect of the future research.

## Supplementary Information

**Additional file 1: Figure S1**. A, Representative image of 3I (scale bar: 50 μm). B, Representative image of 3J. C, Representative image of 3K (scale bar: 50 μm). D, Representative image of 3L. E, Representative image of 3M.

**Additional file 2: Figure S2**. A, Representative image of 4G (scale bar: 50 μm). B, Representative image of 4H. C, Representative image of 4I (scale bar: 50 μm). D, Representative image of 4J (scale bar: 50 μm). E, Representative image of 4K.

**Additional file 3: Figure S3**. A, Representative image of 5A. B, Representative image of 5B. C, Representative image of 5C (scale bar: 50 μm). D, Representative image of 5D. E, Representative image of 5E (scale bar: 50 μm). F, Representative image of 5F (scale bar: 50 μm). G, Representative image of 5G.

**Additional file 4: Figure S4**. A, Representative image of 6F. B, Representative image of 6G (scale bar: 50 μm). C, Representative image of 6H (scale bar: 50 μm).

**Additional file 5: Table S1**. Primer sequences for RT-qPCR. **Table S2**. Correlation analysis between HDAC3 expression and clinicopathological characteristics of patients with ESCC.

**Additional file 6: Table S2**. Correlation analysis between HDAC3 expression and clinicopathological characteristics of patients with esophageal squamous cell carcinoma.

## Data Availability

Data sharing not applicable to this article as no datasets were generated or analysed during the current study.

## Abbreviations

HDACs: Histone deacetylases
ESCC: Esophageal squamous cell carcinoma
TGIF1: Transforming growth factor beta-inducing factor 1
TGFβ: Transforming growth factor-β
GEO: Gene Expression Omnibus
IgG: Immunoglobulin G
HRP: Horseradish peroxidase
cDNA: Complementary DNA
GAPDH: Glyceraldehyde-3-phosphate dehydrogenase
HEEC: Human esophageal epithelial cell
DMSO: Dimethyl sulfoxide
NC: Negative control
DMEM: Dulbecco’s modified Eagle’s medium
PBS: Phosphate buffer saline
FITC: Fluorescein isothiocyanate
PI: Propidium iodide
FBS: Fetal bovine serum
ECM: Extracellular matrix
3’UTR: 3’Untranslated region
ANOVA: Analysis of variance
RT-qPCR: Reverse transcription quantitative polymerase chain reaction
OS: Overall survival
Sh: Short hairpin RNA
MMP: Matrix metalloprotein-2
EdU: Ethynyl-2'-deoxyuridine
ChIP: Chromatin immunoprecipitation
NOVA1: Neuro-oncological ventral antigen 1
WT: Wild-type
MUT: Mutant type
Oe: Overexpression
KEGG: Kyoto encyclopedia of genes and genomes
