# Supplementary material for: Silencing of histone deacetylase 3 suppresses the development of esophageal squamous cell carcinoma through regulation of miR-494-mediated TGIF1
Source: Cancer Cell Int. 2022 May 16;22:191. doi: 10.1186/s12935-022-02581-3 (PMC9109300; doi:10.1186/s12935-022-02581-3)
Supplement: Supplementary file 5 — Additional file 5: Table S1. Primer sequences for RT-qPCR. Table S2. Correlation analysis between HDAC3 expression and clinicopathological characteristics of patients with ESCC. [file 12935_2022_2581_MOESM5_ESM.docx]

**Supplementary Table 1** Primer sequences for RT-qPCR

| Target | Primer sequence (5' - 3') |
| --- | --- |
| miR-494 | F: ATTGAAACATACACGGGAAAC |
|  | R: Universal primer sequence |
| U6 | F: ACAGAGAAGATTAGCATGGCC |
|  | R: Universal primer sequence |
| HDAC3 | F: CGTCCGAAATGTTGC |
|  | R: GAAGTTCCTCACTAATGG |
| TGIF1 | F: ATTCCCTTGGACCTTTCTTC |
|  | R: GCATTGTAACGGTGCTCATA |
| GAPDH | F: AACGGATTTGGTCGTATTGGG |
|  | R: TCGCTCCTGGAAGATGGTGAT |

Note: RT-qPCR, reverse transcription quantitative polymerase chain reaction; miR-494, microRNA-494; HDAC3, histone deacetylase 3; TGIF1, transforming growth factor beta-inducing factor 1; GAPDH, glyceraldehyde-3-phosphate dehydrogenase; F, forward; R, reverse.

**Supplementary Table 2** Correlation analysis between HDAC3 expression and clinicopathological characteristics of patients with ESCC

| Clinicopathological characteristics | n | HDAC3 expression | *p* |
| --- | --- | --- | --- |
| Age (years) |  |  | 0.374 |
| < 55 | 38 | 2.426 ± 0.349 |  |
| ≥ 55 | 41 | 2.509 ± 0.464 |  |
| Gender |  |  | 0.949 |
| Male | 43 | 2.472 ± 0.414 |  |
| Female | 36 | 2.466 ± 0.415 |  |
| Tumor size |  |  | < 0.001 |
| < 5 cm | 63 | 2.363 ± 0.35 |  |
| ≥ 5 cm | 16 | 2.886 ± 0.38 |  |
| TNM staging |  |  | < 0.001 |
| I | 46 | 2.335 ± 0.286 |  |
| II | 21 | 2.406 ± 0.479 |  |
| III | 12 | 3.017 ± 0.248 |  |
| Lymph node metastasis |  |  | 0.002 |
| Present | 19 | 2.721 ± 0.433 |  |
| Absent | 60 | 2.389 ± 0.375 |  |

Note: ESCC, Esophageal squamous cell carcinoma; HDAC3, histone deacetylase 3; TNM, tumor node metastasis.
